# Supplementary material for: ROS- and pH-Responsive Polydopamine Functionalized Ti3C2Tx MXene-Based Nanoparticles as Drug Delivery Nanocarriers with High Antibacterial Activity
Source: Nanomaterials (Basel). 2022 Dec 9;12(24):4392. doi: 10.3390/nano12244392 (PMC9786132; doi:10.3390/nano12244392)
Supplement: Supplementary file 1 [file nanomaterials-12-04392-s001.zip › nanomaterials-2076931-supplementary.pdf]

nanomaterials

Supplementary Materials

ROS- and pH-Responsive Polydopamine Functionalized $Ti_3C_2T_x$ MXene-Based Nanoparticles as Drug Delivery Nanocarriers with High Antibacterial Activity

Wei-Jin Zhang [1], Shuwei Li [2], Veena Vijayan [3,4], Jun Seok Lee [1], Sung Soo Park [5], Xiuguo Cui [6], Ildoo Chung [1], Jaejun Lee [1], Suk-kyun Ahn [1], Jung Rae Kim [2], In-Kyu Park [3,4] and Chang-Sik Ha [1,*]

[1] Department of Polymer Science and Engineering, School of Chemical Engineering, Pusan National University, Busan 46241, Republic of Korea
[2] School of Chemical Engineering, Pusan National University, Busan 46241, Republic of Korea
[3] Department of Biomedical Sciences, Chonnam National University Medical School, Gwangju 61469, Republic of Korea
[4] BioMedical Sciences Graduate Program (BMSGP), Chonnam National University, Hwasun 58128, Republic of Korea
[5] Division of Advanced Materials Engineering, Dong-Eui University, Busan 47340, Republic of Korea
[6] School of Material Science and Engineering, Beijing Institute of Petrochemical Technology, Beijing 102617, China
* Correspondence: csha@pnu.edu

Thioglycolic acid was reacted with acetone to form a thioketal compound diacetoxyl thioketal.

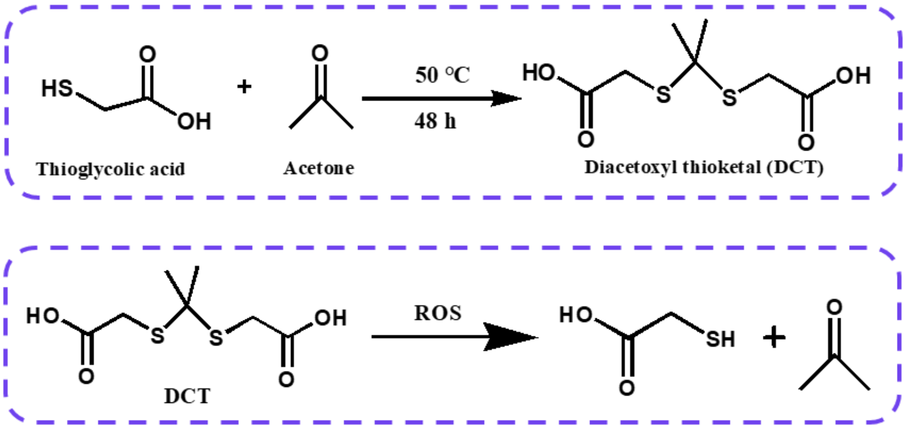

Figure S1. Synthesis of ROS-cleavable diacetoxyl thioketal (TK) linker.

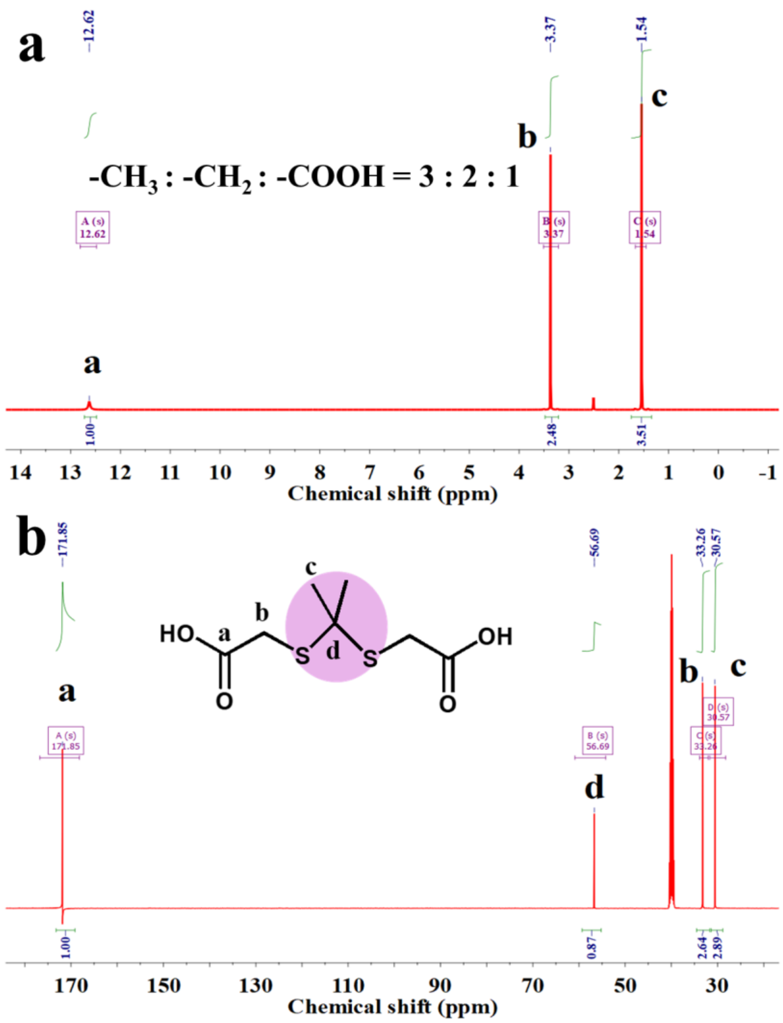

Figure S2. NMR spectra of TK ROS-cleavable linker in DMSO-d6 (**a**) ^{1}H and (**b**) ^{13}C.

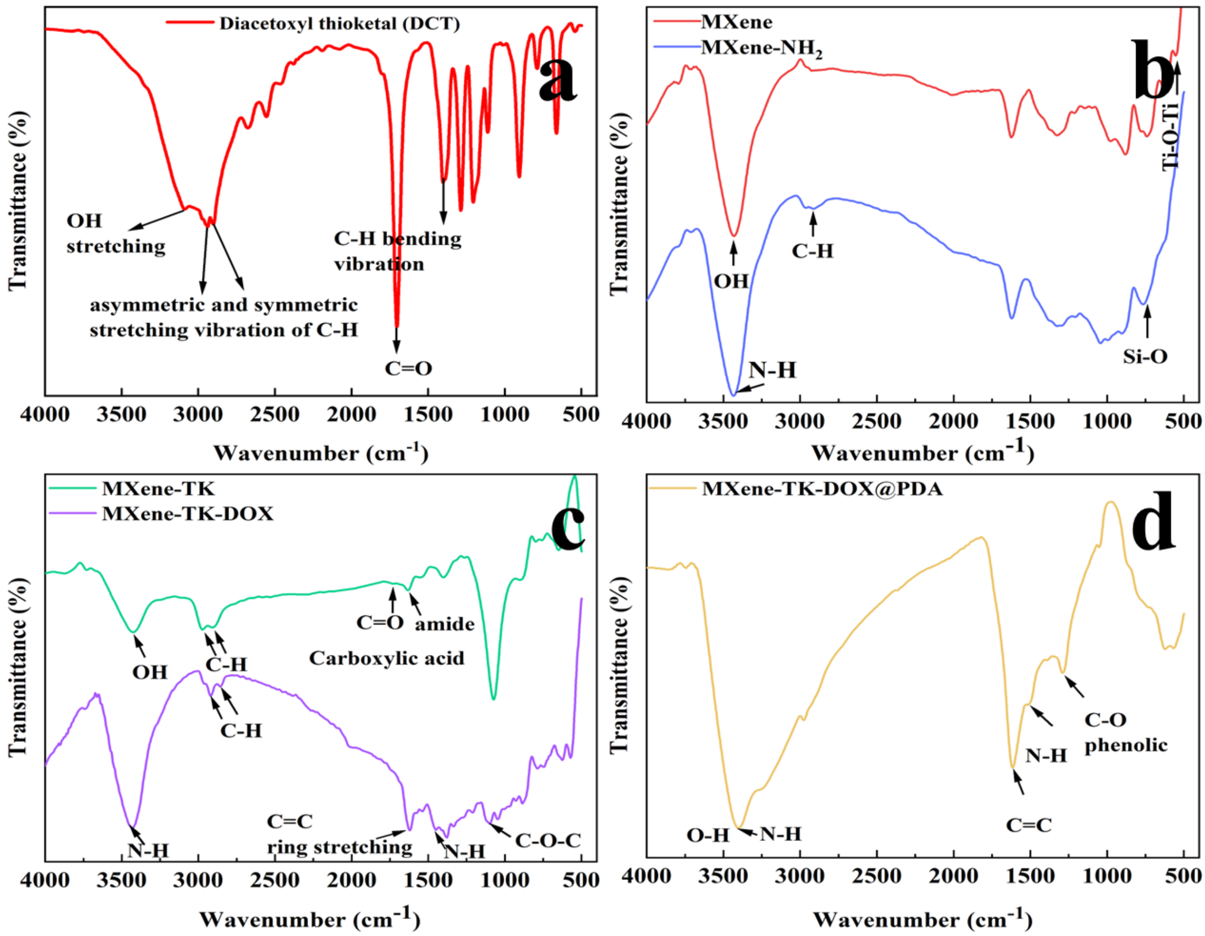

Figure S3. FTIR spectra analysis. (**a**) The ROS-cleavable TK linker, (**b**) MXene and MXene−NH_2 nanosheets, (**c**) MXene−TK and MXene−TK−DOX flaks, (**d**) MXene−TK−DOX@PDA nanoparticles.

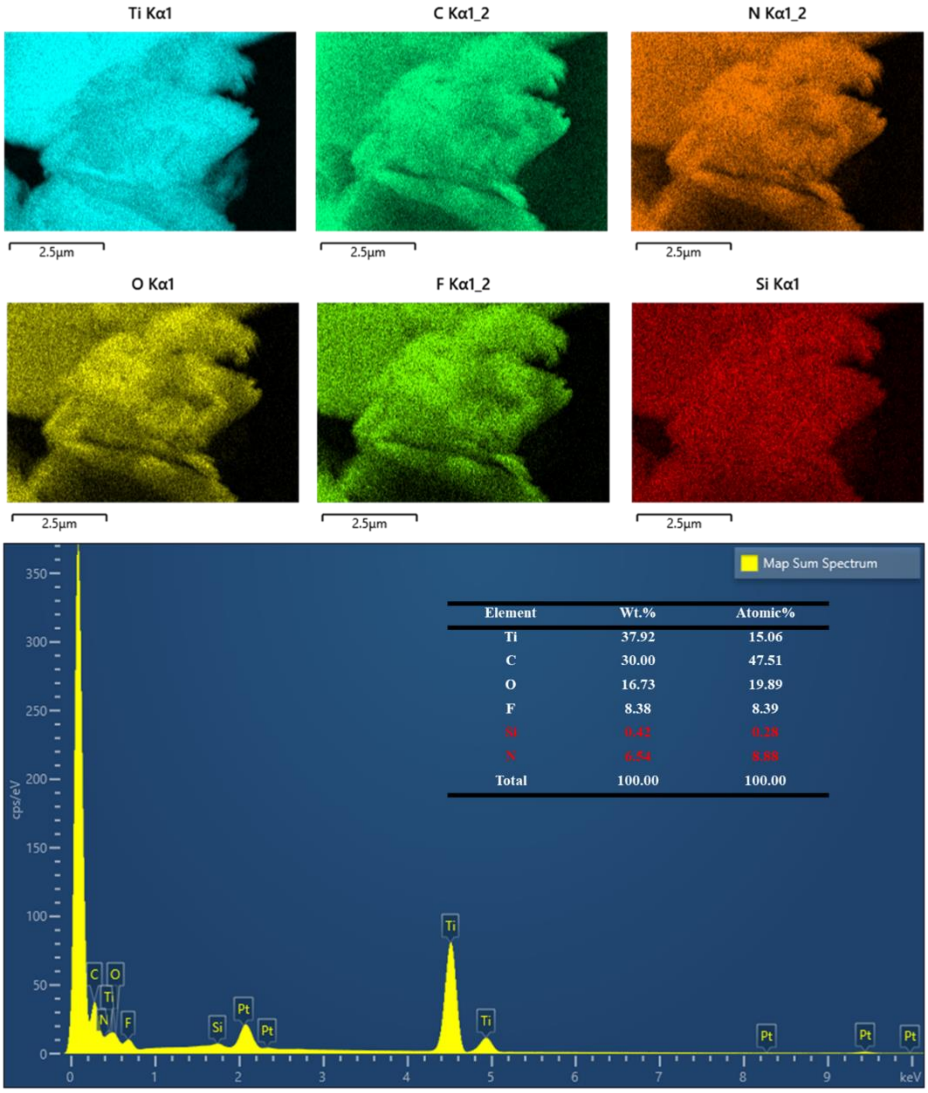

Element	Wt.%	Atomic%
Ti	37.92	15.06
C	30.00	47.51
O	16.73	19.89
F	8.38	8.39
Si	0.42	0.28
N	6.54	8.88
Total	100.00	100.00

Figure S4. EDX (mapping) of MXene–NH_2 nanosheets.

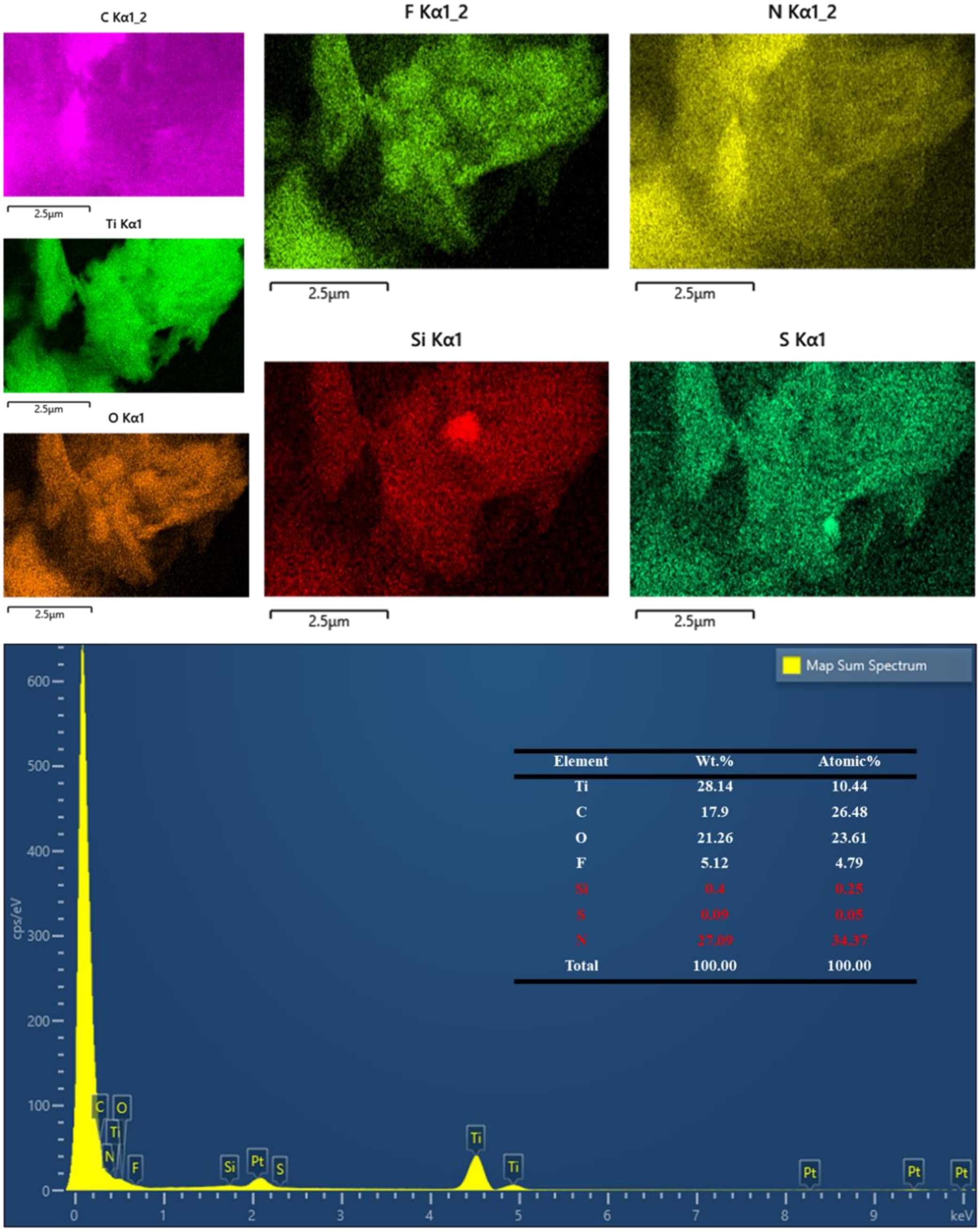

Element	Wt.%	Atomic%
Ti	28.14	10.44
C	17.9	26.48
O	21.26	23.61
F	5.12	4.79
Si	0.4	0.25
S	0.09	0.05
N	27.09	34.37
Total	100.00	100.00

Figure S5. The EDX (mapping) of MXene–TK nanosheets.

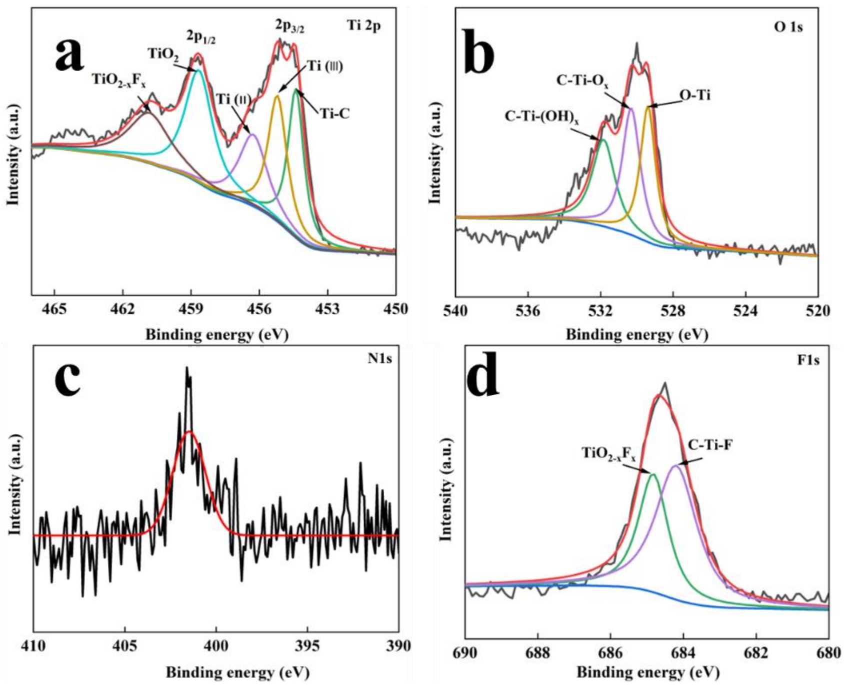

Figure S6. XPS spectrum of MXene nanosheets after delamination by TBAOH. (**a**) O1s, (**b**) Ti 2p, (**c**) F1s, and (**d**) N1s.

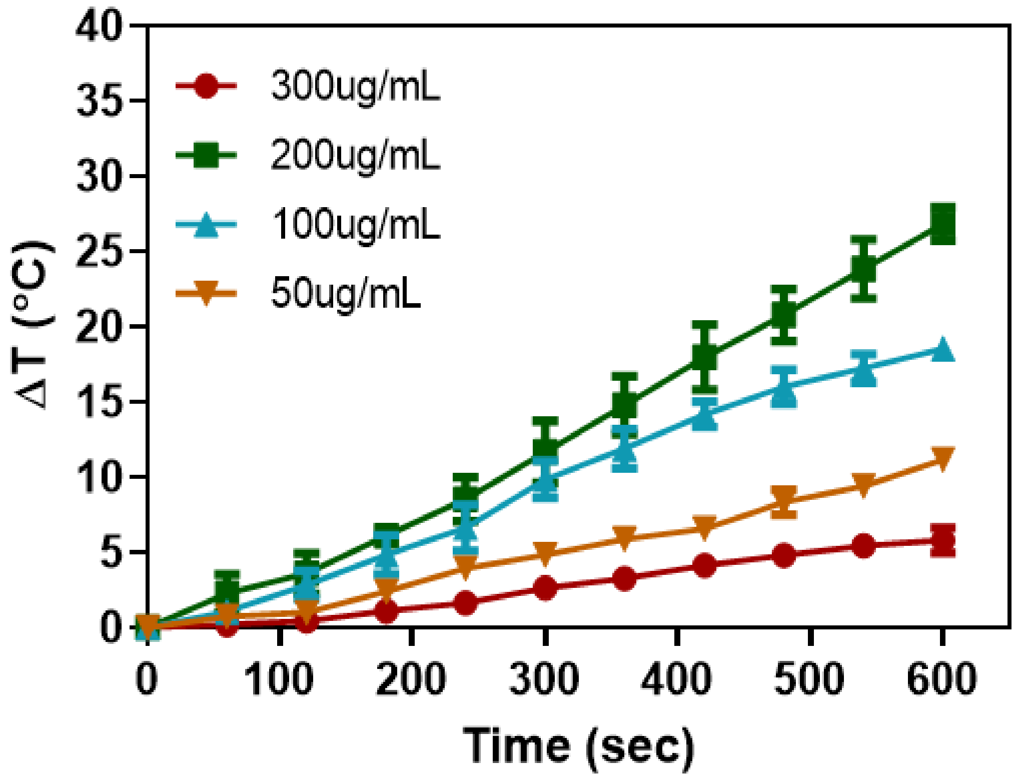

Figure S7. Photothermal-heating profile of MXene aqueous solution after 808 nm laser irradiation (2 W/cm^2) at elevated concentrations (50, 100, 200, and 300 ug/mL).

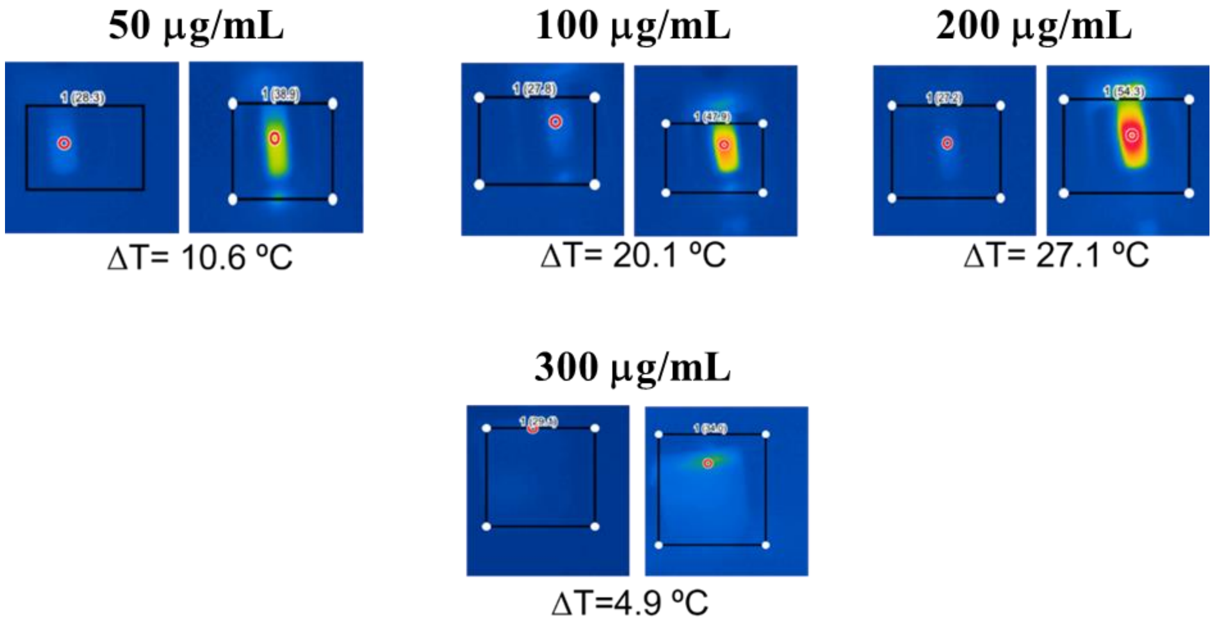

Figure S8. Infrared thermal images of MXene nanosheets irradiated with 808 nm laser (2 W/cm[2]) for 10 min at varied concentrations (50, 100, 200, and 300 ug/mL).

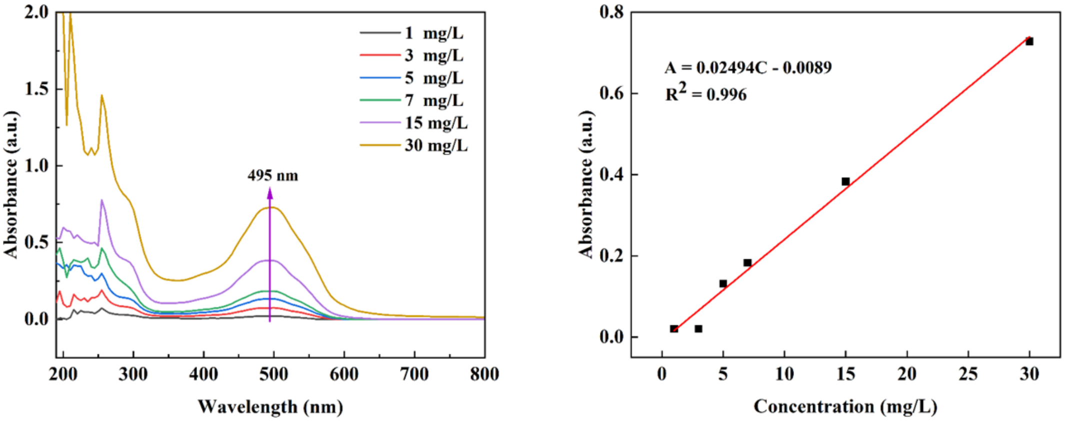

Figure S9. Calibration curve of doxorubicin in PBS solution.

Table S1 In vitro doxorubicin release kinetics study for different formulations based on the Higuchi model.

Sample Code	Release Medium	Higuchi	
		K_h*	R^2
MXene-TK-DOX@PDA	pH 5.5	2.299	0.976
	pH 7.4	0.071	0.879
	pH 7.4 + H_2O_2	5.993	0.995
	pH 5.5 + H_2O_2	7.931	0.993

K_h* represents the release rate constant, and R^2 represents the correlation coefficient.

The general simplified Higuchi model equation is expressed as follows.

Higuchi: $$M_t = k_h t^{1/2} \quad (1)$$

Where M_t is the release rate at time t and k_h is the rate constant.
